# Lightweight Carbon–Metal-Based Fabric Anode for Lithium-Ion Batteries

**DOI:** 10.1021/acsami.4c01601

**Published:** 2024-04-17

**Authors:** Barun Kumar Chakrabarti, Gerard Bree, Anh Dao, Guillaume Remy, Mengzheng Ouyang, Koray Bahadır Dönmez, Billy Wu, Mark Williams, Nigel P. Brandon, Chandramohan George, Chee Tong John Low

**Affiliations:** † Sabancı Üniversitesi Nanoteknoloji Araştırma ve Uygulama Merkezi (SUNUM), Orta Mahalle Üniversite Caddesi No:27, 34956 Tuzla, Istanbul, Turkey; ‡ WMG, Warwick Electrochemical Engineering Group, Energy Innovation Centre, University of Warwick, Coventry CV4 7AL, U.K.; § Centre for Imaging, Metrology, and Additive Technology (CiMAT), WMG, University of Warwick, Coventry CV4 7AL, U.K.; ∥ Department of Earth Science and Engineering, Imperial College London, London SW7 2AZ, U.K.; ⊥ Dyson School of Design Engineering, Imperial College London, London SW7 2AZ, U.K.

**Keywords:** lithium-ion battery, carbon−metal fabric, electrospinning, free-standing electrode, current collector

## Abstract

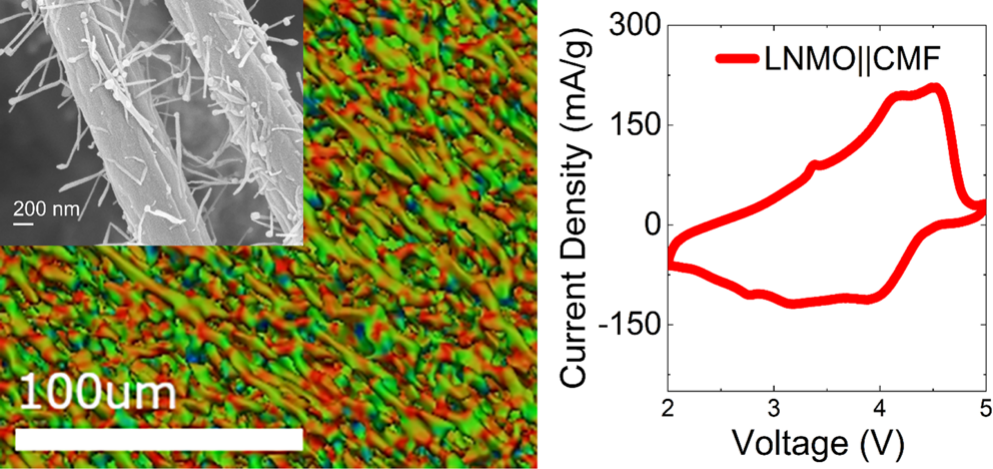

Lithium-ion battery electrodes are typically manufactured via slurry casting, which involves mixing active material particles, conductive carbon, and a polymeric binder in a solvent, followed by casting and drying the coating on current collectors (Al or Cu). These electrodes are functional but still limited in terms of pore network percolation, electronic connectivity, and mechanical stability, leading to poor electron/ion conductivities and mechanical integrity upon cycling, which result in battery degradation. To address this, we fabricate trichome-like carbon–iron fabrics via a combination of electrospinning and pyrolysis. Compared with slurry cast Fe_2_O_3_ and graphite-based electrodes, the carbon–iron fabric (CMF) electrode provides enhanced high-rate capacity (10C and above) and stability, for both half cell and full cell testing (the latter with a standard lithium nickel manganese oxide (LNMO) cathode). Further, the CMFs are free-standing and lightweight; therefore, future investigation may include scaling this as an anode material for pouch cells and 18,650 cylindrical batteries.

## 1 Introduction

Since their commercialization in 1991 by Sony Corp., the energy density of lithium-ion batteries has almost tripled (<300 Wh·kg^–1^) with significant cost reduction (≤100 $·kWh^–1^).^1^ With cathode chemistries (e.g., NMC, NCA, LFP, LMO)^2^ diversified, the choice of anodes^3^ is still limited to mostly graphite, while silicon and lithium titanium oxide (LTO) are secondary choices, and metallic lithium is under development. However, despite graphite being the most commonly used anode, its low capacity (∼372 mAh·g^–1^) and propensity to form lithium–metal dendrites is problematic.^4^ Silicon has a high theoretical specific capacity (4200 mAh·g^–1^), but its volume expansion (over 300%) leads to particle cracking as a result of Si–Li alloying reactions that limit the use of pure silicon.^5^ Commercial applications use carbon-rich Si composites to achieve trade-offs between stability and capacity, while LTO has low conductivity and limits the nominal full cell voltage as it has higher lithiation voltages (∼1.5 V vs Li/Li^+^).^6^

Conversely, conversion-type anodes, such as metal oxides,^7^ offer high capacity (∼1000 mAh·g^–1^ for iron oxide-based materials), but their voltage hysteresis and poor reversibility impede further progress.^8^ One of the main reasons why such conversion-type electrodes are not practically employed in lithium-ion batteries is because traditional electrode processing via slurry casting results in electrodes with poor lifetimes.^9^ This arises from the way in which slurry casting is performed, which typically produces electrodes with active particles mixed with a polymer binder (PVDF = poly(vinylidene fluoride)) and carbon additives in a high boiling point solvent (NMP = *N*-methyl-2-pyrrolidone), which forms an ink to be cast onto current collectors and dried. This often leads to poor control of the electrode microstructures and, in certain cases, severe mechanical instability. However, the slurry casting method, whether using NMP or water as solvent, works much better for intercalation materials (e.g., graphite, LFP), where the volume expansion issues (or stress induced breaking of active particles) are relatively minimal on each successive cycle but still not fully suitable for long-term cycling.^10^

Another attractive alternative to slurry casting for battery electrodes involves electrospinning followed by carbonization, which were used to produce free-standing plain nonwoven carbon fabric electrodes successfully.^11^ Such electrospun fabrics exhibited more than double the volumetric capacity upon cycling beyond several hundred times, in some cases, compared to slurry cast electrodes, which could be attributed to better electrical contact with the active particles via the carbon fiber network. These carbon fabrics were then either decorated^12^ with battery active particles or with active particles grown^13^ directly on the carbon fibers or tubes. While these hybrid electrodes had become more functional, their microstructural features were still not conducive to support lithium-ion battery performances at less than 500 cycles at high C-rates due to detachment of active particles from carbon fibers and excessive electrolyte decomposition, thereby increasing internal resistance.^14^ Recently, we reported a novel electrode architecture resembling trichomes consisting of micron-sized carbon fibers, carbon nanotubes (CNTs), and Fe nanoparticles based on electrospinning and pyrolysis (termed as carbon–metal fabric or CMF), demonstrating their advantageous performance in a Zn-air battery as free-standing electrodes.^15^ The trichome-like electrodes were also employed as carbon catalyst layers for hybrid hydrogen/vanadium flow cells successfully, albeit being coupled with graphene-modified carbon paper/cloth materials to endure the harsh operating conditions of high flow rates (50–100 mL·min^–1^) and current densities (100 mA·cm^–2^) in the constant presence of 5 M sulfuric acid.^16^ In spite of such promising performances in the aforementioned energy storage devices, understanding the behavior of these trichome-like morphologies in lithium-ion battery applications, compared to traditional slurry cast electrodes, remains a research gap. In this work, we therefore present the electrochemical characteristics of our CMF electrodes on Li ion battery cycling at a relatively high C-rate due to multiple advantages attributable to their ion/electron conductivity and mechanical integrity when compared to their counterparts prepared by means of standard slurry casting.

## 2 Experimental Section

### 2.1 Materials

*N*,*N*′-dimethylformamide (DMF, technical grade for electrospinning, ∼94% pure) was purchased from VWR International. Polyacrylonitrile (PAN) powder was purchased from Goodfellow Cambridge Limited Huntingdon (average particle size ∼50 μm, molecular weight ∼230,000 g·mol^–1^). Iron(III) acetylacetonate, Fe(acac)_3_, was purchased from Sigma-Aldrich (≥97%). For lithium-ion coin cell testing, the following electrode materials were used: synthetic graphite powder (PGPT350, Targray), iron oxide or Fe_2_O_3_ (powder, <5 μm, ≥96%, Sigma-Aldrich), lithium iron phosphate or LFP (ALEEES A14), and lithium nickel manganese oxide or LNMO (spinel, powder, <0.5 μm particle size (Brunauer–Emmett–Teller (BET)), >99%, Sigma-Aldrich).

### 2.2 Synthesis of Carbon–Metal Fabric Electrodes

To prepare the CMF trichome electrodes, Fe(acac)_3_ powder was added in ratios of 40 wt % relative to PAN and mixed for 24 h at 55 °C as described elsewhere.^16^ The as-prepared precursor solution was then transferred to a syringe and pumped into the electrospinning needle at 1.5 mL·h^–1^ by a syringe pump integrated with an electrospinning machine (Bioinicia LE-50). High voltages of 10–15 kV were applied onto the needle (25 mm length, 20 G) to extract the fiber at a stable rate, with a working distance of 15 cm from the grounded rotating collector, tightly covered by a layer of Al foil, and with a rotating speed of 1500 rpm. The electrospinning was done at 25 °C and at 50% humidity. The as-prepared free-standing nanofiber film was peeled off from the Al collector for subsequent heat-treatment. The electrospun fiber films were calcined in air at 280 °C for 2 h for the stabilization process, followed by a pyrolysis step at 850 °C in nitrogen (N_2_) with a 2 °C·min^–1^ ramp rate.

### 2.3 Material Characterization

Scanning electron microscopy (SEM) was performed by using a Zeiss Sigma microscope with a coupled Oxford Instruments energy dispersive X-ray spectroscopy (EDS) detector. EDS was conducted and analyzed using Aztec software.

X-ray diffraction (XRD) was conducted using an Aeris desktop machine with data analysis performed by means of Highscore software (Malvern Panalytical). To understand the electrochemical trend observed in terms of electrode microstructures, we performed an X-ray computerized tomography (XCT) analysis of CMF electrodes. The CMF sample was scanned on a ZEISS Vectra 520 for determining the surface microstructures of interest. The specimen was scanned at a voltage of 80 kV, a power of 7 W, an exposure time of 20 s, and a minimum of 10× over 1601 projections as reported previously.^16^

### 2.4 Slurry Cast Electrode Preparation

As controls, graphite, Fe_2_O_3_, and LFP and LNMO electrodes were produced via slurry casting. Graphite powder was weighed out along with conductive carbon black (CB, Imerys C65) and binder additives like styrene–butadiene rubber (SBR, Zeon) and sodium carboxymethyl cellulose (CMC, Ashland) at a ratio of 91:5:2:2 graphite/CB/SBR/CMC. The mixture was dispersed in deionized water at a solid content of 35% using a high viscosity centrifugal mixer (Thinky ARE-310) to form a slurry. The Fe_2_O_3_, LFP, and LNMO slurries were produced using formulations of 90:5:5 of active material/PVDF/CB, dispersed in *N*-methyl pyrrolidone (NMP) at a solid content of 43%. The PVDF utilized was Solef 5130 (Solvay). Anode slurries (graphite or Fe_2_O_3_) were cast onto 9 μm Cu foils (MTI) while cathode slurries were cast onto 15 μm Al foils (MTI). Anode thicknesses were controlled to provide loadings similar to the CMF, while cathode thicknesses were chosen to achieve a full cell N/P ratio of approximately 1:1. Electrodes produced with the water-based slurry were dried overnight at 60 °C in air, while those produced with the NMP-based slurries were placed in a vacuum oven at 120 °C overnight. The dried electrodes were subjected to a calendaring step to increase the active layer’s adhesion and density.

### 2.5 Electrochemical Characterization

The electrodes were tested in coin-type cells (2032), in both half cell and full cell configurations. Fifteen mm discs were punched (using an EL-punch) from the electrodes, and the coin cell was assembled by using an electric crimper. In both cases, a two-electrode setup was employed for the coin cells (i.e., an additional reference electrode was not used). For half cell studies, the CMF functioned as working electrodes while 250 μm Li foil discs (Cambridge Energy Solutions) were employed as counter and reference electrodes. For full cell studies, LFP or LNMO served as the cathode while the CMF was utilized as the anode. 120 μL of electrolyte (1 M LiPF_6_ in 1:1 ethylene carbonate/diethyl carbonate) was added to the cells prior to crimping. The completed cells were mounted onto a Biologic BCS cycler inside a temperature-controlled oven at 25 °C and allowed to soak for 10 h before completing 3 formation cycles at a rate of C/20. The cycling program and data analysis was performed using BT Lab software. Cyclic voltammetry (CV), galvanostatic cycling, and electrochemical impedance spectroscopy (EIS) were then performed.

The galvanostatic intermittent titration (GITT) test involved the application of a C/10 charge/discharge current for 30 min, alternating with 30 min rest periods, during which no current was applied (Figure S1, Supporting Information). This approach enabled calculation of the lithium diffusion coefficient within the material, using the equation^17^
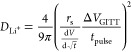
where *D*_Li_^+^ is the lithium diffusion coefficient, *r*_s_ is the particle diameter, *V* is the cell voltage, *t* is the time, Δ*V*_GITT_ is the difference in steady state voltage before and after the current pulse, and *t*_pulse_ is the pulse time. *D*_Li_^+^ for the CMF was calculated over the full state of charge (SOC) range over the course of a (de)lithiation cycle.

## 3 Results and Discussion

### 3.1 Morphological Characterization of CMF Electrodes

**Figure 1:**
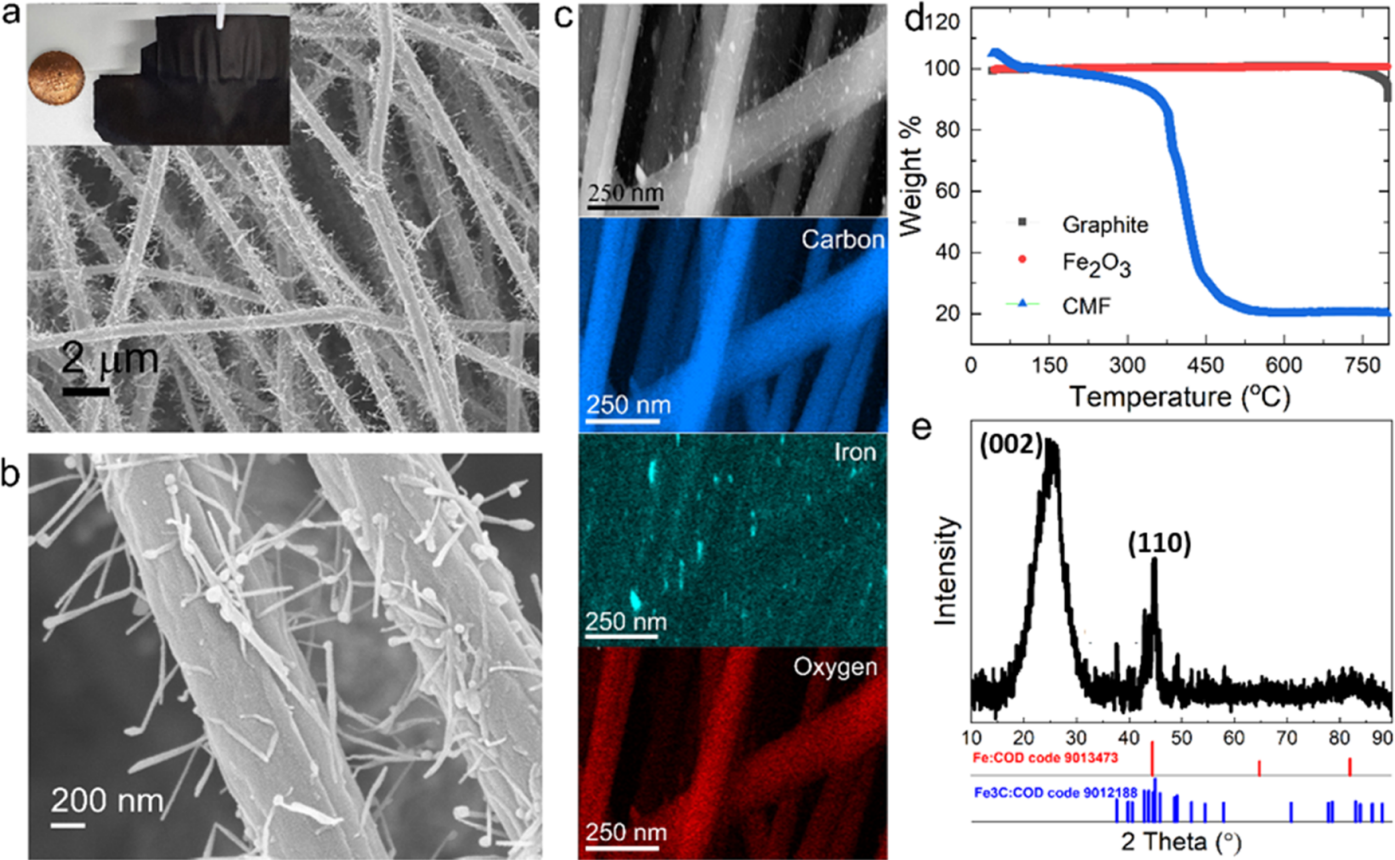
(a) A scanning electron microscopy image of carbon–Fe trichomes (CMF) and a photo of the CMF (inset); (b) close-up of fibers in CMFs, where CNTs with iron catalyst particles can be seen; (c) SEM of a few carbon microfibers along with SEM-energy dispersive X-ray chemical mapping of CMFs at 250 nm; (d) thermogravimetric analysis of CMFs and different electrode slurries processed via traditional slurry casting as controls; (e) XRD of the pristine CMF (Fe: COD 9013473 and Fe3C: COD code 9012188).

Figure 1a,b shows SEM images of the trichome-like carbon–Fe fabrics used as electrodes at different magnifications (individual carbon fiber with CNTs and a few carbon fibers), where the inset shows a photo of the free-standing CMF (as against a British penny). Micron-sized carbon fibers mostly run in parallel directions (SEM images; see Figure 1), which form the basic skeleton for the free-standing fabrics. The Fe-based nanoparticles are not only embedded in micron-sized carbon fibers because of their growth process but also catalyze the growth of CNTs,^15^ which assemble directly on the micron-sized carbon fibers. The Fe catalyst particles can be located either at the tip of the CNTs in some cases (tip-growth), which indicates that during carbonization of fibers, weakly interacting nanoparticles were lifted off, or at the bottom, where the ones firmly adhering to the fibers caused base growth of the fibers as the carbonization temperature was increased (Figure 1b). The SEM image shown in Figure 1b reveals carbon nanotubes grown from the surface of the carbon fiber. SEM-energy dispersive X-ray spectroscopy (EDX) chemical mapping confirms the presence of well-distributed carbon and Fe throughout the carbon fibers as in Figure 1c. Figure S2 (Supporting Information) shows a photograph of the CMF along with three-dimensional (3D) reconstructed images of the same electrode at a size of 50 μm (where fibers are clearly visible). Figure S3 shows that the CMF can be successfully bent to a 45° angle without sustaining any material loss. The main purpose of Fe nanoparticles was to catalyze the growth of CNTs on CMFs to obtain a trichome-like morphology rather than a battery active material; however, the subsequent exposure to air during handling of CMFs may cause some oxidation of Fe particles at a surface level, which may show some electrochemical activity in batteries, but to a negligible extent.

Figure 1d shows a thermogravimetric (TGA) analysis of the CMF carried out in air compared with slurry cast samples of Fe_2_O_3_ (iron oxide) and graphite electrodes. Material loss is normalized to that at 100 °C, with any values below this temperature being attributed to adsorbed water. The pure Fe_2_O_3_ samples do not lose mass up to 800 °C, while the CMF thermally decomposes to approximately 20% of its initial mass. The mass loss was attributed to the carbon component, and thus it was concluded that the CMF consists of approximately 80:20 carbon/Fe by weight. This ratio can be easily adjusted by the initial concentrations of polyacrylonitrile (PAN) binder and Fe-based (iron acetylacetonate) precursor as reported by some of us elsewhere.^15^ The synthesis conditions for the CMF are detailed in Section 2.

Figure 1e shows the XRD patterns of pristine CMF samples (comparison with cycled samples is shown in a subsequent figure). The obtained X-ray diffractogram validates the X-ray mapping analyses. The peak around approximately a 2θ value of 25° corresponds to the structured carbon nanomaterial. On the other hand, the peak around a 2θ value of 44° is attributed to Fe atoms at the base of the micron-sized carbon fibers and to some extent to those present at the tips of CNTs (COD code 9013473). The extent of graphitization of the carbon material can be determined through an examination of the (002) carbon peak. Nongraphitic carbon has an interlayer spacing of 0.344 nm, while for fully graphitized samples the value is 0.3354 nm. The graphitization value can therefore be expressed as^15^
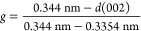
*d*(002) was calculated using Bragg’s Law: *n*λ = 2*d* sin(θ), where λ is the wavelength of incident radiation (0.15406 nm), *n* is the order of reflection (1), *d* is the interplane spacing, and θ is the angle of reflection. The graphitization value for the pristine CMF was 36%. The degree of graphitization shifts the hybridization in the carbon fiber structure from sp^3^ to sp^2^. This leads to an improvement in the electron transfer capability. Thus, it is expected that the increased conductivity due to the graphitization degree and Li diffusion contribute to the capacity and energy density, while the rapid access of ions contributes to the power density.

### 3.2 Electrochemical Studies in Coin Cells

**Figure 2:**
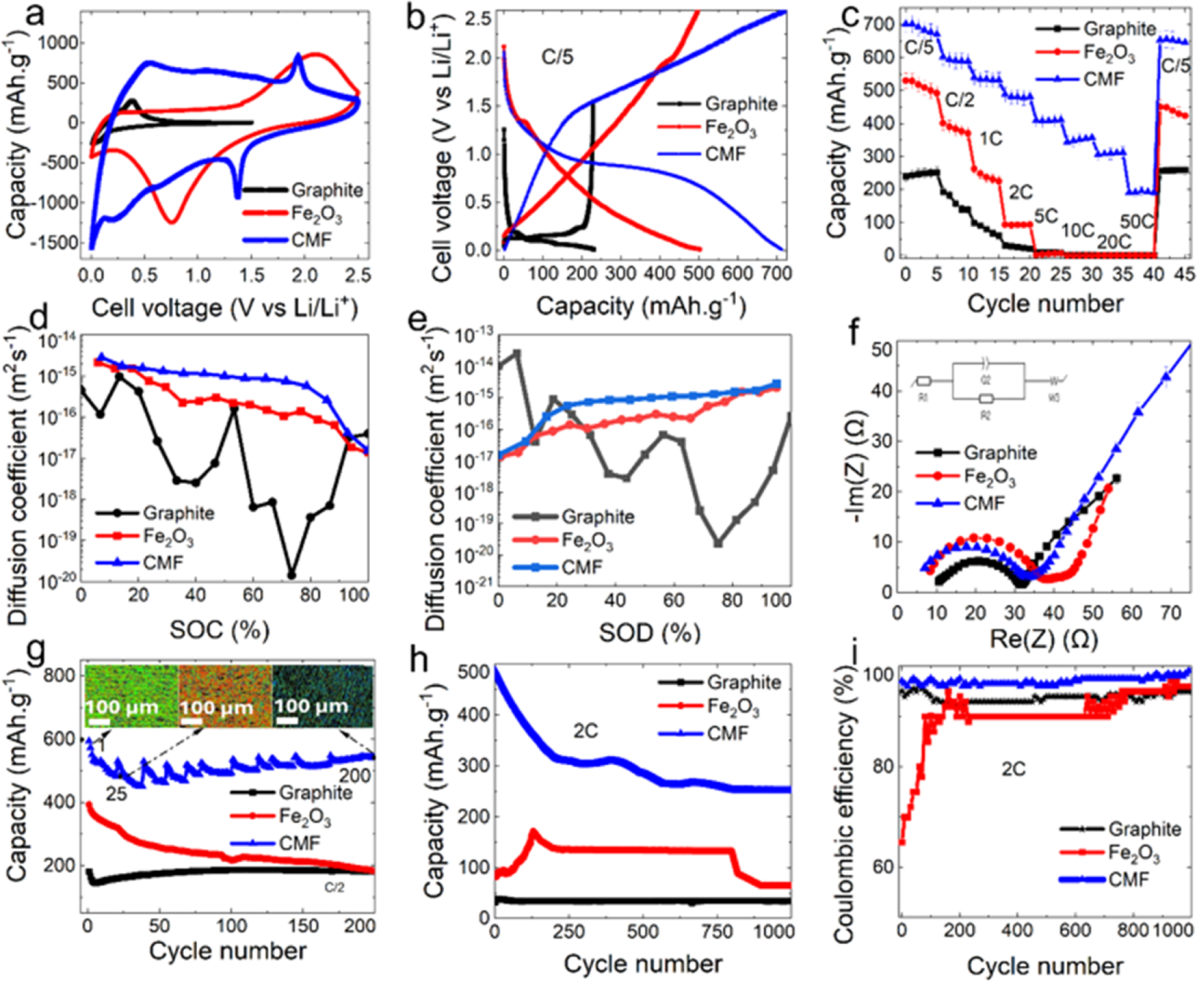
(a) CV of the CMF anode compared with slurry cast control counterparts at 10 mV·s^–1^; (b) voltage profile of coin cells using either the CMF or slurry cast anodes at C/5; (c) rate capability test of CMFs and control electrodes; (d) Li ion diffusion coefficient of CMFs and control electrodes processed by slurry casting obtained from GITT curves for state of charge (SOC); (e) Li ion diffusion coefficient obtained from GITT for state of discharge; (f) resistivities of CMFs and the controls by means of EIS; (g) long-term cycling of CMFs and controls at C/2 rate: CMF fiber structures at three different cycles (1, 25, and 200) are shown from the X-ray microcomputed tomography (XCT) reconstructed images within the plot itself (in these images, colors represent the fiber orientations whereby green is horizontal, blue is vertical, and red is diagonal, respectively); (h) long-term cycling up to 1000 times of Li ion coin half cells at 2C using three different anodes; (i) Coulombic efficiencies for cycling Li ion coin cells up to 1000 times, showing excellent stability of CMF anodes at a high C-rate.

The performance of the CMFs was then assessed in lithium-ion battery half cells (with lithium–metal in 2032 coin-type cells). The details of the cell fabrication and testing procedures can be found in Section 2. Cyclic voltammetry (CV) was performed on the CMF (utilizing lithium-foil as both the counter and reference electrodes) (Figure 2a). Comparing the CV responses of the CMFs and controls (graphite- or iron oxide-based electrodes prepared by conventional slurry casting), the CMFs show lithiation/delithiation processes that fall between those of graphite and iron oxide. The CMF exhibits anodic and cathodic peaks at 0.5 and 0.25 V vs Li/Li^+^, attributed to Li^+^ intercalation/deintercalation into/from the graphitic carbon (Li + C_6_ ↔ LiC_6_). Very small peaks around 1.9 V (anodic) and 1.4 V (cathodic) were observed, and these could be related to the conversion process corresponding to 6Li + Fe_2_O_3_ ↔ 3Li_2_O + 2Fe, which are most likely due to the lithiation of some surface oxides. Although the CMFs show sharper peaks (∼1.4 and 1.9 V vs Li/Li^+^) related to (de)lithiation processes of the oxides, this is negligible because the shape of the CV curves shows a more capacitive behavior from CMFs compared to both graphite- and iron oxide-based electrodes, indicating that the capacity is mainly due to carbon–lithium reactions. It is also observed that the slurry cast Fe_2_O_3_ anode exhibits a broad oxidation and reduction peak, while in the CMF material, as expected, these peaks have shifted and narrowed. Furthermore, the nature of the CMF material inherently results in a parallelogram-shaped voltammogram. The carbon fibers with CNTs in the CMF architecture appear to contribute to this promising performance, particularly at high C-rates. The capacitive nature of the voltammogram obtained with the CMF is due to the controlled porous structure of the CMF and graphitization, which is favorable to promoting ion transport, accommodating volume change, facilitating interfacial charge transfer, and improving electrolyte penetration.^18^ In addition, upon scrutinizing the voltammograms of all three samples, it is evident that the CMF material is poised to deliver maximum performance, and the synergy between the morphological features and material combination significantly influences both the capacity and C-rate capability.

The voltage profiles of the CMFs and the control (iron oxide-based electrodes prepared by conventional slurry casting) show that CMFs achieve higher capacities despite the negligible contribution from the Fe-related surface oxide phase. The profile of the CMF (vs Li/Li^+^) indicates that the capacity of the CMF mainly comes from a carbon–Li intercalation phenomenon and to a negligible extent from Fe_2_O_3_–Li due to surface oxidation of Fe nanoparticles in CMFs (see Figure 2b). This is in line with Li intercalation studies reported in the literature for PAN-derived hard carbon.^19^ With the slurry cast Fe_2_O_3_ anode, a broad oxidation is clearly observed during charging, and a corresponding broad reduction reaction of the same nature is evident during discharge. When examining the charge–discharge curve of the CMF material, it is observed that the voltages at which these reactions occur shift, indicating a change in its capacitive behavior. The capacity of the CMFs was found to be competitive given the mass loading (CMFs, Fe % compared to the Fe_2_O_3_ slurry cast electrode, Fe %). Additionally, the rate capacity tests show that CMFs achieve a balance in terms of rate and capacity (Figure 2c).

The initial capacity obtained for the CMF material at a rate of C/5 is approximately 700 mAh·g^–1^. In contrast, for an anode material containing only Fe_2_O_3_, this is around 530 mAh·g^–1^. The main advantage of the CMF electrodes not only constitutes an increase in capacity but is also associated with an enhanced current density, indicating the superior C-rate capability. While the Fe_2_O_3_ electrodes show significant capacity loss with increasing current density, the capacity loss in the CMF electrodes is markedly lower. Additionally, at high C-rates (5, 10, 20, and 50C), Fe_2_O_3_ electrodes fail to function, whereas the CMF material achieves a high discharge capacity, which adds weight to the argument that the capacitive nature of carbon fibers with CNTs in the CMF structure contributes to the performance.

The galvanostatic intermittent titration testing was carried out on the CMF, slurry cast Fe_2_O_3_, and graphite electrodes to compare the lithium diffusion characteristics of the materials. The results suggest a better lithium diffusivity across CMFs compared with the Fe_2_O_3_ slurry cast electrode. This can translate into better rate capability at high current charge/discharge, as shown above (Figure 2c). Values were compared with those of a standard graphite electrode measured in the same way (Figure 2d,e). At SOCs > 25%, the CMF exhibited better diffusion coefficients than the graphite material. At low SOCs (<25% lithiation), the reverse was the case. A lower lithium diffusivity (*D*_Li_^+^) is expected for graphite at higher SOCs as Li^+^ must diffuse into already populated sites in the graphite. This effect is reduced with the CMF due to the capacitive contribution observed from the cycling data and also as described in the literature.^20^ Li diffusion is also sluggish in graphite grain boundaries, which is overcome via the CMF electrodes.^21^

Notably, for the CMF, there was a significant difference in *D*_Li_^+^ values between the lithiation and delithiation phases at high levels of lithiation. This was because at high levels of lithiation, graphitic intercalation/deintercalation was the dominant process in the CMF. Deintercalation of lithium from graphite was typically faster than intercalation.^22^ The ohmic polarization of the cells was determined by examining the step change in cell voltage observed immediately upon the application of a pulse current, also known as the IR drop. Similar values for polarization were observed for the CMF and graphite electrodes for SOC ≤ 80%; however, at higher levels of SOC, the CMF material exhibited far lower polarization than graphite.

From the impedance data (Figures 2f and S4), the CMFs exhibit charge transfer resistance comparable to that of the control electrodes but lower series resistance (corresponding to the *x*-axis intercept of the impedance curves at the high frequency range). In the case of the CMF, a single semicircle was visible, in the medium frequency region. Conversely, the graphite spectrum revealed the presence of two semicircles, which may be attributed to the resistance associated with the charge transfer process within the solid electrolyte interphase (SEI) layer (high frequency) and the active material (medium frequency). Given the operational voltage of the CMF electrode, the presence of an SEI would be expected; however, its contribution to the resistance was not resolvable from that of the active material itself. The CMF electrode exhibited a significantly lower total resistance (31.9 Ω) than the slurry cast graphite (71.5 Ω) and iron oxide electrodes (45 Ω). This can be attributed to the advantageous morphology of the fibrous material in the CMF, providing a high surface area while maintaining long-range channels for charge transfer. The components calculated as a result of fitting the Nyquist plot obtained by EIS measurements with the equivalent circuit shown in Figure 2f were presented in Table S1. The enhanced charge transfer kinetics was consistent with the better performance of the CMF at high cycling rates (Figure 2c). Additionally, up to 200 cycles could be performed at C/2 that displayed better performance of the CMF in comparison to the control samples (Fe_2_O_3_ and graphite-based electrodes) as shown Figure 2g. Longer-term cycling at 2C displayed an exceptional Li ion coin cell discharge capacity for the CMF (Figure 2h) along with excellent and consistent Coulombic efficiencies (Figure 2i). The main reason for such performances of the CMF was clearly not to do solely with the presence of negligible surface iron oxide or the carbonaceous material present in it but also due to the impact of carbon nanotubes that formed on its surface after undergoing carbonization (CNT formation was catalyzed by the presence of iron nanoparticles present in the noncarbonized electrospun mat). These results were consistent with our previous study on the application of CMFs as electrode materials for zinc-air batteries as reported by Liu et al.^15^

Nevertheless, despite some capacity loss over 200 cycles with this material (for cycling at 2C), it is worth noting that the capacity offered by this material between 200 and 1000 cycles is higher than those of the other two-electrode active materials prepared via the slurry casting method. The anode containing Fe_2_O_3_ exhibits an initial increase in capacity at high C-rates (Figure 2h). This phenomenon has been discussed in the literature for two main reasons. First, the increase in surface area due to electrochemical milling leads to an increase in capacity,^23^ and second, high-rate lithiation-induced activation processes,^24^ which can explain the observed increase up to a certain number of cycles.

**Figure 3:**
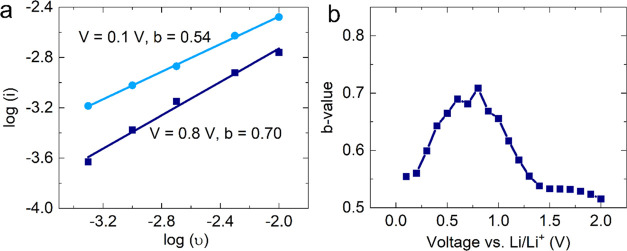
Faradaic and capacitive contributions of a CMF electrode. (a) *b*-Values for each voltage for the CMF are calculated from the slope of log(υ) vs log(i); shown as examples are the fittings for *V* = 0.1 and 0.8 V vs Li/Li^+^. (b) *b*-Value as a function of voltage vs Li/Li^+^.

Given a relatively high surface area of the CMF with trichomes (118.6 m^2^·g^–1^ with trichomes as compared to 35.5 m^2^·g^–1^ without trichomes),^15^ it seems likely that there is a significant contribution to the capacity from a capacitive mechanism associated with Li^+^ adsorption onto its surface. Furthermore, both carbon and iron oxide are established supercapacitor electrode materials.^25^ The specific contributions of energy storage mechanisms can be differentiated through an analysis of the current response of the electrode to a variation of the voltammetric sweep rate. The current obeys a simple power law:^26^
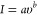
where *I* is the current, υ is the scan rate, and *a* and *b* are constants. A *b*-value of 0.5 corresponds to a faradaic mechanism, while a value of 1 indicates capacitive behavior. A CMF half cell was subjected to cyclic voltammetry, utilizing scan rates in the range 0.5–10 mV·s^–1^. The *b*-values were extracted by plotting log(*I*) against log(υ), with the examples of 0.1 and 0.8 V as shown in Figure 3. *b*-Values corresponding to the lithiation of the CMF electrode are shown in Figure 3a,b. With observed *b*-values close to 0.5, the faradaic mechanism is dominant, particularly so in the low voltage (<0.5 V) and high voltage (>1.5 V) regions. This is consistent with the dual stage lithiation of iron oxide (>1.5 V) followed by that of graphite (<0.5 V). Nevertheless, the significant contribution from capacitance is clearly visible and probably enhances the high current capability of the CMF electrode.

### 3.3 XCT and XRD Analysis of Pristine and Cycled CMF Electrodes

**Figure 4:**
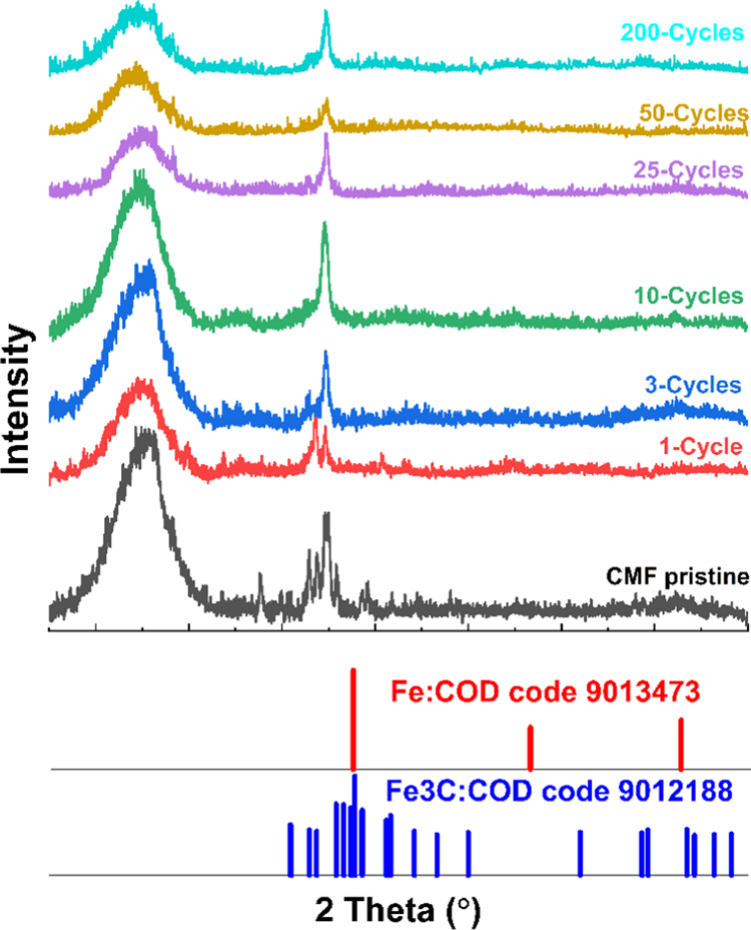
X-ray diffraction patterns of a CMF electrode upon cycling.

**Figure 5:**
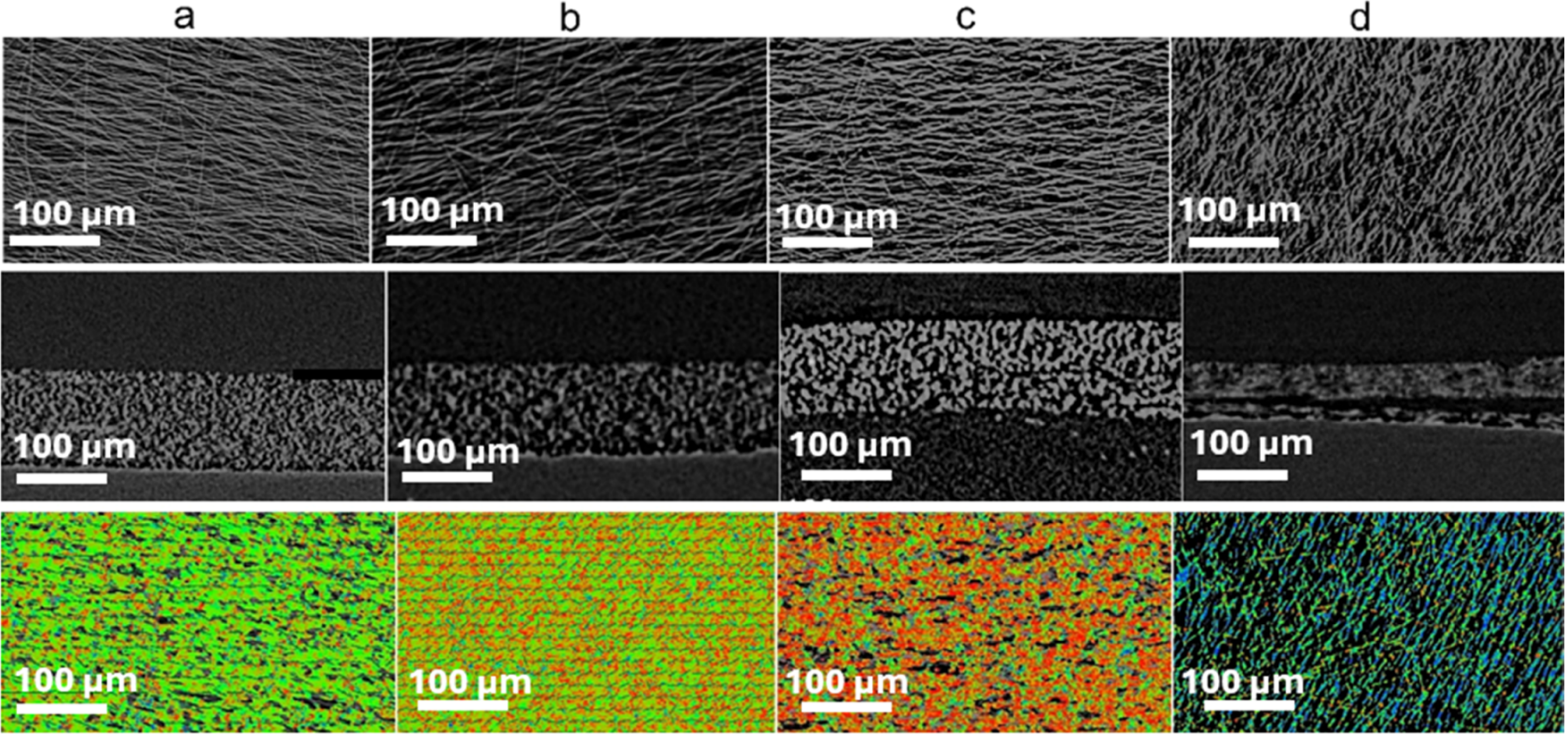
X-ray computed tomography (XCT) images of the CMF electrode before and after cycling at C/2; (a) pristine CMF top view, side view, and fiber orientation; (b) CMF after cycle 1, top view, side view, and fiber orientation; (c) CMF after 25 cycles, top view, side view, and fiber orientation; (d) CMF after 200 cycles, top view, side view, and fiber orientation.

The CMF samples were then characterized by X-ray diffraction (XRD) and tomography (XCT) to observe the changes before and after cycling at C/2. XRD patterns of the CMFs corresponding to different cycling stages are shown in Figure 4, which is in line with CV and SEM-EDX results in terms of the fact that the presence of Fe gives a minor or negligible contribution to the capacity (i.e., capacity of CMFs is predominantly derived from carbon reactions, similar to graphite but not exactly the same mechanism as that of graphite possibly due to the presence of CNTs). In Figure 5, XCT images (at 680 nm) show CMF electrodes at different cycling stages, where the top view, side view, and alignment of carbon fibers in CMFs are compared. The fibers and Fe metal domains can easily be observed, and for the case of the early charge/discharge cycles, there is not much structural distortion, while the areas (lumps in Figure 5c) that appear brighter are due to the distribution of high-density materials. In the 3D image, green (horizontal), blue (vertical), red (diagonal) represent the fiber orientations. Brighter areas (points) are basically high-density metal clusters, as the whole structure (scaffold) appears to be integrated into one solid lump (after reaction with Li^+^). Pore structures are more easily definable with sizes of ∼180 nm and above, and these structures feature more noticeable pores and networks (after pore analysis via XCT).

As the XRD pattern of the bare CMF is examined in Figure 4, it is evident that a mixed diffractogram from two different materials is obtained. Following a cycle of charge and discharge of the cell, a decrease in the intensity of peaks from both materials is observed. With the continued cycle number (first 25 cycles), a significant decrease in peaks attributed to Fe persists. On the other hand, this phenomenon also explains the capacity loss observed for the CMF material in the first 25 cycles. Normally, this capacity loss is more pronounced in an electrode containing only Fe_2_O_3_ (slurry cast). Nevertheless, the negligible surface-based Fe_2_O_3_ in the CMF structure continues to provide some electroactivity, and after 25 cycles, the CMF still maintains the highest specific capacitance, completing the 200-cycle test with an overall increasing trend (Figure 2g). This situation is indicative of the carbon fiber–Fe structure serving as a good host for Li.

Upon examination of the XCT image of the CMF in Figure 5a,b, a slight thinning of the electrode is observed, indicating good compatibility between the electrode and the electrolyte, with the fibers naturally experiencing volume loss within the electrolyte. Additionally, Figure 5a,b showcases distinct crystal orientations of carbon fibers in the CMF structure. As the cycle number increases, a change in the crystal structure of the electrode is observed, as shown in Figure 5c. Still, the robust nature of the CMF is evident in the side view of the XCT image, indicating that, despite changes in the chemical structure of the electrode, the carbon skeleton remains undegraded.

Further examination in Figure 5d reveals that even after 200 cycles, the CMF electrode does not show degradation, although some morphological change such as cracks is evident in the side-view XCT image. These findings align with the results of the 200-cycle charge–discharge test for the CMF depicted in Figure 2g.

### 3.4 Full Cell Studies with LNMO

**Figure 6:**
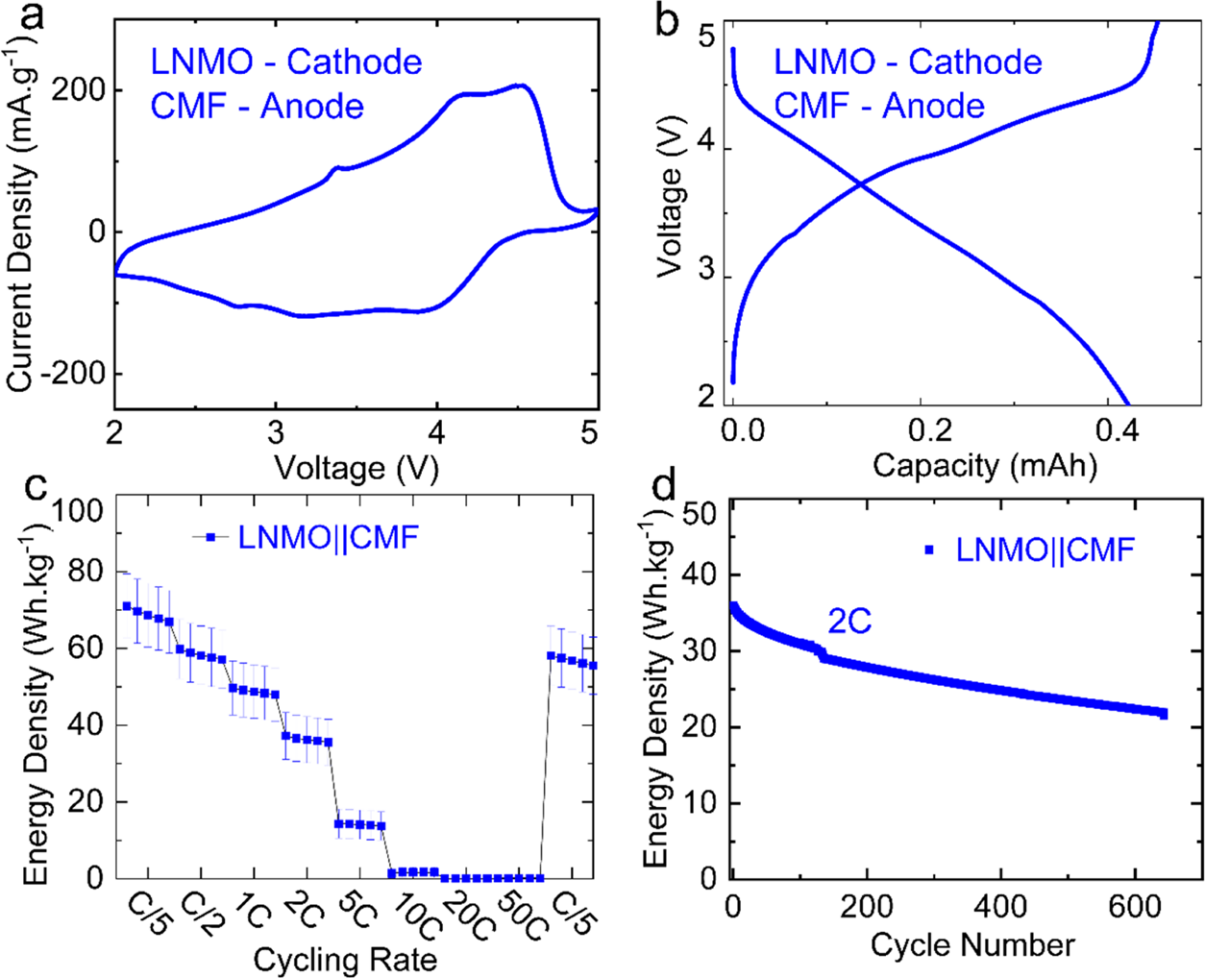
Electrochemical performance of a CMF in a full cell with a LNMO cathode. (a) CV of LNMO/CMF cells at 10 mV·s^–1^; (b) voltage profile at C/2; (c) rate capability results; (d) energy density retention of LNMO/CMF full cells upon cycling at 2C.

Finally, we evaluated the CMFs in full commercial-style coin cells containing LNMO as the cathode material (processed via slurry casting). Figure 6 shows the data from the LNMO full cells. The LNMO||CMF combination is particularly advantageous as this cathode material enables a good cell voltage (average discharge voltage is 3.70 V) that consequently results in a higher energy density.^27^ As can be seen from Figure 6a, it is evident that the oxidation peaks occurring in the range 4–4.75 V correspond to the oxidation reactions of Ni^2+^/Ni^3+^ and Ni^3+^/Ni^4+^. The broad reduction peak observed at approximately 4 V encompasses the redox reactions of Ni^4+^/Ni^3+^ and Ni^3+^/Ni^2+^. The peaks occurring in the range of approximately 2.5–3.5 V are attributed to the transformations of Mn^2+^/Mn^3+^.^28^ The obtained cyclic charge and discharge curves (Figure 6b) corroborate the cyclic voltammogram, confirming that the CMF anode operates well with the LNMO cathode. This is also evident from the rate capability and cycling results shown in Figure 6c,d. The LNMO||CMF full cell also demonstrates reasonable capacity retention while undergoing repeated cycling at 2C (Figure 6d). It is expected that an optimization of the electrolyte for the CMF will further enhance the full cell performance. Furthermore, the amount of Fe particles (which are required to cause trichome-like growth) can be reduced, and the recipe can be extended to cathode particles integrated in the CMFs. Comparative results of using a CMF as an anode with LNMO or LFP cathode materials are given in the Supporting Information (Figure S5).

### 3.5 Manufacturing and Costing Considerations

The robustness and free-standing nature of CMF electrodes are crucial as it removes the need for an inactive current collector. On the anode side of a lithium-ion battery, the current collector typically consists of a heavy (∼10 μm) copper foil, onto which the graphite slurry is cast. To elucidate the true advantage of the CMF, the capacity of the half cells is calculated as a function of the total electrode mass (i.e., including current collector foil) and is shown in Table 1. Therefore, in the analyses and capacity calculations, the total mass of both the current collector and the active material has been taken into consideration for each active material. When this is considered, the CMF provides a large capacity advantage of 720%.

**Table 1:** Comparison of a Stand-Alone CMF Electrode’s Specific Capacity as against Slurry Cast Electrodes

material	active layer loading (mg·cm^–2^)	capacity/active layer mass (mAh·g^–1^)	foil loading (mg·cm^–2^)	total loading (mg·cm^–2^)	capacity/total electrode mass (mAh·g^–1^)
graphite	2.6	221	8.9	11.5	49
Fe_2_O_3_	3.0	529	8.9	11.9	133
CMF	2.0	402	0	2.0	402

The potential weight savings in a commercial cell was examined through analysis of a hypothetical 18650 cylindrical lithium-ion battery (with an LFP cathode) utilizing a CMF in place of the graphite-on-Cu anode (LFP full cell results are shown in Figure S5 of the Supporting Information). The masses of the various cell components were taken from a previous study^29^ and are shown in Table 2. The higher capacity of the CMF material compared with graphite reduced the required anode active mass and crucially enabled the elimination of the Cu foil (approximately 10% of the cell mass). This brought about a 12.8% reduction in total cell mass, while maintaining the same gravimetric capacity.

**Table 2:** Typical Component Masses of an 18,650 Cell

		mass (g)	
component	composition	graphite	CMF
cathode coating	LFP	9.66	9.66
anode coating	graphite or CMF	5.18	4.05
cathode current collector	aluminum	2.14	2.14
anode current collector	copper	3.86	0
electrolyte	Li salt in a carbonate mixture	6.41	6.41
separator	polypropylene/polyethylene	1.15	1.15
cell casing	stainless steel	10.45	10.45
total cell mass (and energy density)		38.85	33.86 (70 Wh·kg^–1^)^a^

a It is the energy density calculated based on the specific capacity obtained at a C/5 rate.

## 4 Conclusions

We have presented a new free-standing trichome-like electrode architecture based on the carbon–metal fabric (CMF) for lithium-ion batteries. The electrochemical performance of CMF electrodes together with their morphological stability on cycling shows that these can be a potential replacement for traditional slurry casting methods. Due to the presence of carbon nanotubes that are supported by the presence of Fe nanoparticles, CMFs offer competitive rate capability performances at different C-rates up to 50C in lithium-ion battery half cells. Full lithium-ion battery coin cell testing using LNMO cathode material showed that the energy (related with cell capacity) of the cell dropped by about 40% upon cycling at 2C for 500 times, highlighting their suitability for further optimization. The recipe for preparing CMFs can also be extended to manufacture cathode materials, thus producing all integrated/sandwiched free-standing electrodes, offering improved mass loading and electrical accessibility of energy storage particles. The free-standing nature of these fabrics also makes them better candidates for next-generation mechanically pliable electrodes for ultra flexible batteries.

## Supporting Information Available

Electrochemical evaluation of lithium-ion battery coin cells using CMFs as electrodes are given; both half- and full cell testing results (especially comparing LFP with LNMO cathodes) are shown with some resistance calculations from EIS; results are expanded beyond what has been presented in the main text (PDF)
