# Supplementary material for: Lightweight Carbon–Metal-Based Fabric Anode for Lithium-Ion Batteries
Source: ACS Appl Mater Interfaces. 2024 Apr 17;16(17):21885–94. doi: 10.1021/acsami.4c01601 (PMC11071045; doi:10.1021/acsami.4c01601)
Supplement: Supplementary file 1 — am4c01601_si_001.pdf [file am4c01601_si_001.pdf]

# Supporting Information (SI)

## Lightweight Carbon-metal Based Fabric Anode for Lithium-ion Batteries

Barun Kumar Chakrabarti[1,2,*,a] Gerard Bree[2,a] Anh Dao[2], Guillaume Remy[3], Mengzheng Ouyang[4], Koray Bahadır Dönmez[1], Billy Wu[5], Mark Williams[3], Nigel P Brandon[4], Chandramohan George[5,*] and Chee Tong John Low[2,*]

[1]Sabancı Üniversitesi Nanoteknoloji Araştırma ve Uygulama Merkezi (SUNUM), Orta Mahalle Üniversite Caddesi No:27, 34956, Tuzla – İstanbul, Turkey
[2]WMG, Warwick Electrochemical Engineering Group, Energy Innovation Centre, University of Warwick, Coventry, CV4 7AL, United Kingdom
[3]Centre for Imaging, Metrology, and Additive Technology (CiMAT), WMG, University of Warwick, Coventry, CV4 7AL, United Kingdom
[4]Department of Earth Science and Engineering, Imperial College London, London SW7 2AZ, United Kingdom
[5]Dyson School of Design Engineering, Imperial College London, London SW7 2AZ, United Kingdom

Corresponding Authors: Barun Kumar Chakrabarti (barun.chakrabarti@sabanciuniv.edu, Chandramohan George (Chandramohan.George@imperial.ac.uk), John Low (C.T.J.Low@warwick.ac.uk)

[a] These authors contributed equally to this work.

# GITT results for LIB half-cells

The complete range of results obtained from GITT testing are given in Figure S1.

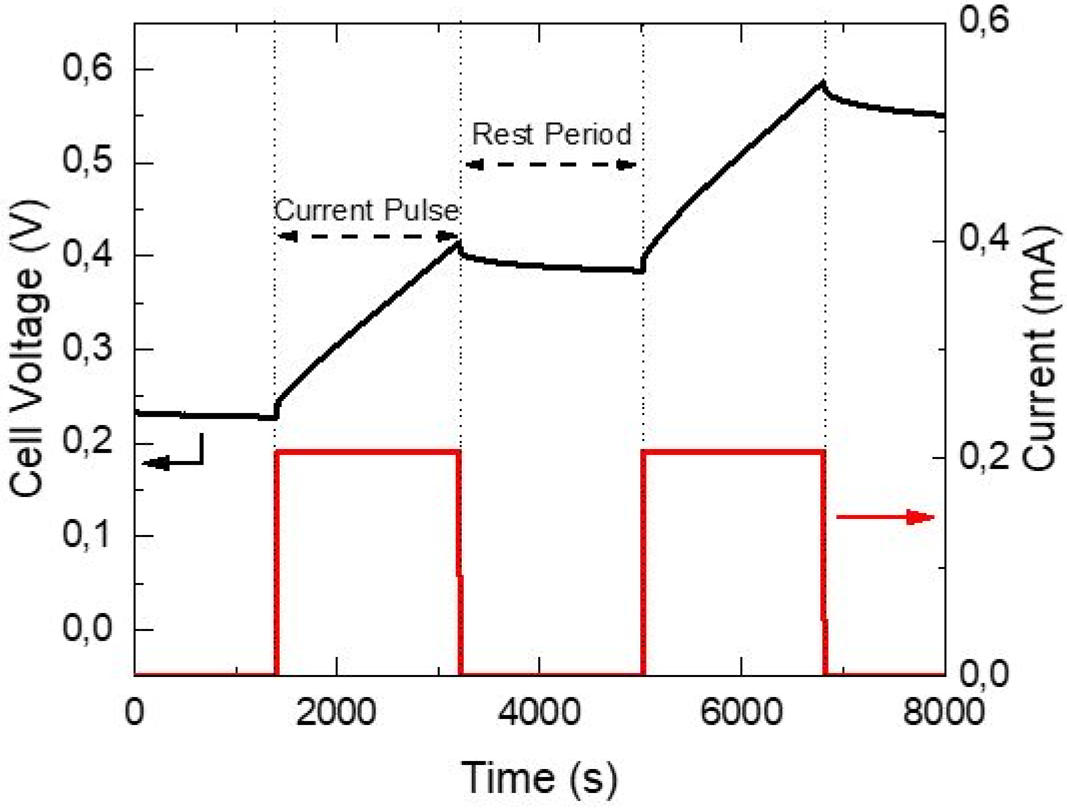

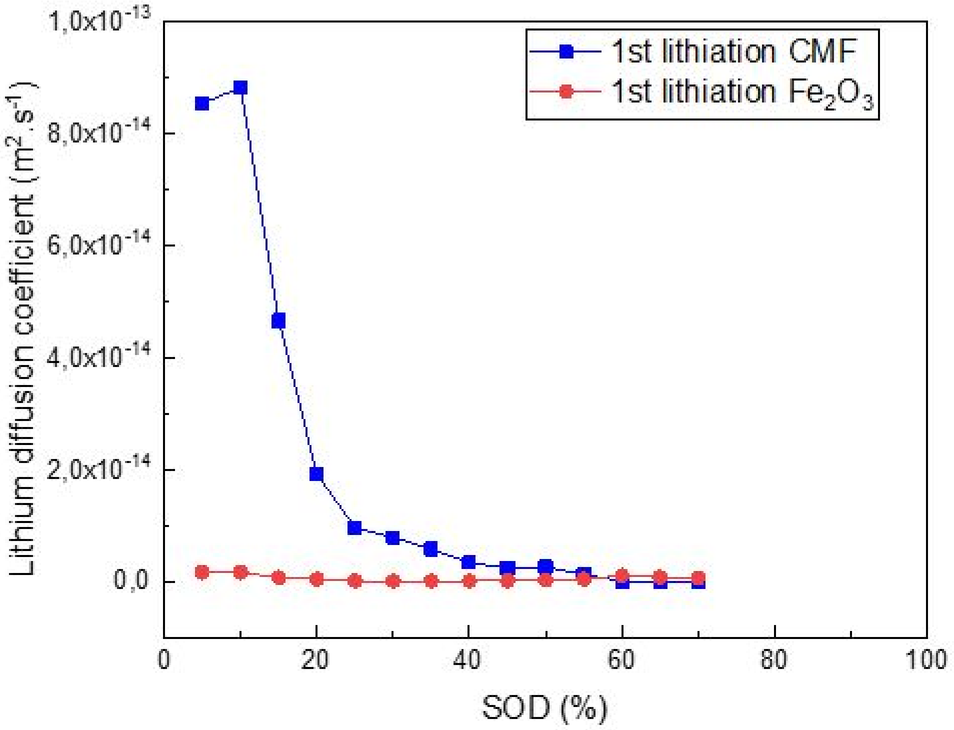

1,0x10-13
8,0x10-14
6,0x10-14
4,0x10-14
2,0x10-14
0,0
Lithium diffusion coefficient (m2.s-1)
0
20
40
60
80
100
SOD (%)
1st lithiation CMF
1st lithiation Fe2O3


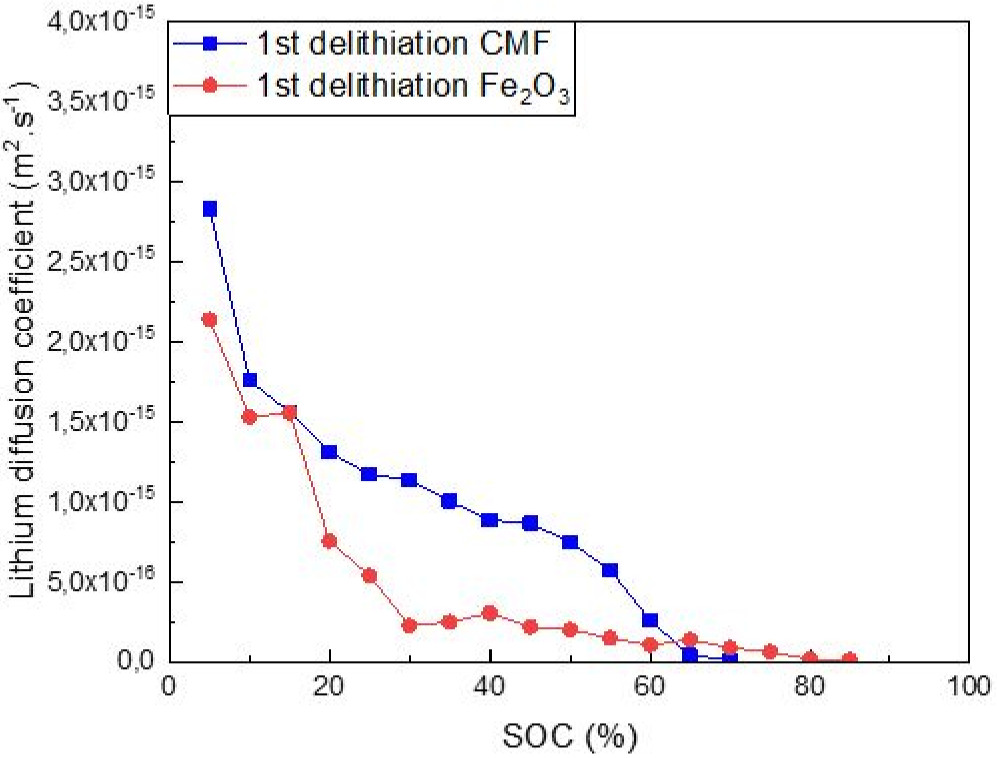

4,0x10-15
3,5x10-15
3,0x10-15
2,5x10-15
2,0x10-15
1,5x10-15
1,0x10-15
5,0x10-16
0,0
Lithium diffusion coefficient (m2.s-1)
0
20
40
60
80
100
SOC (%)
1st delithiation CMF
1st delithiation Fe2O3

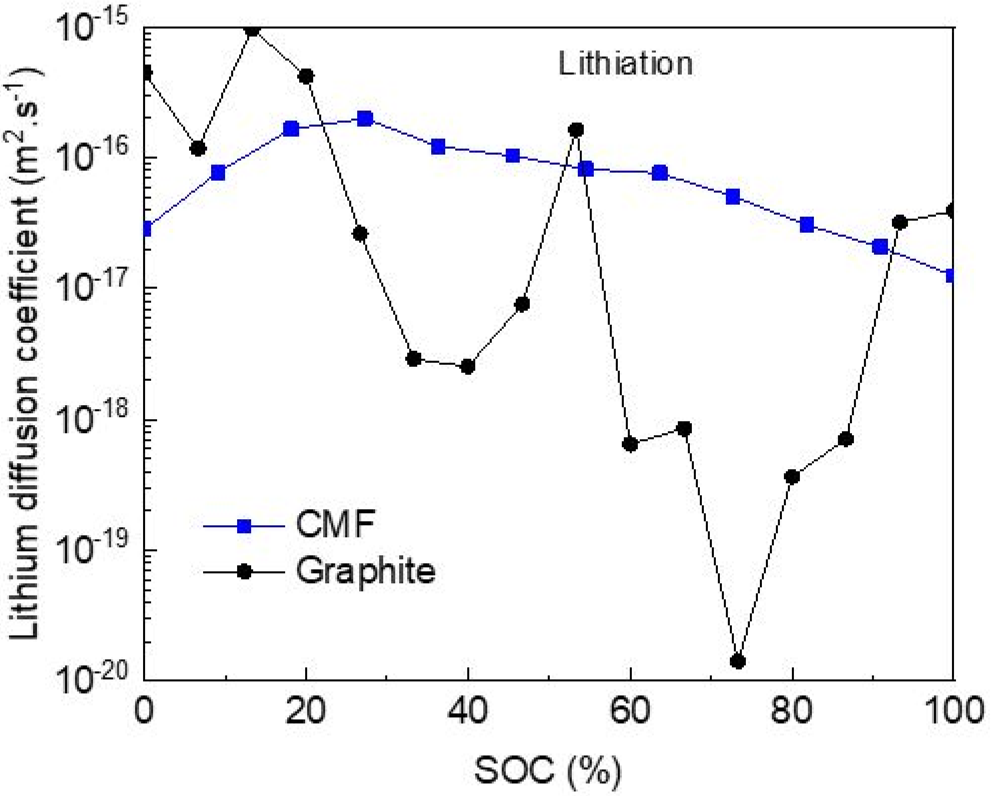
Lithiation
CMF
Graphite
Lithium diffusion coefficient (m2.s-1)
SOC (%)
10-15
10-16
10-17
10-18
10-19
10-20
0
20
40
60
80
100

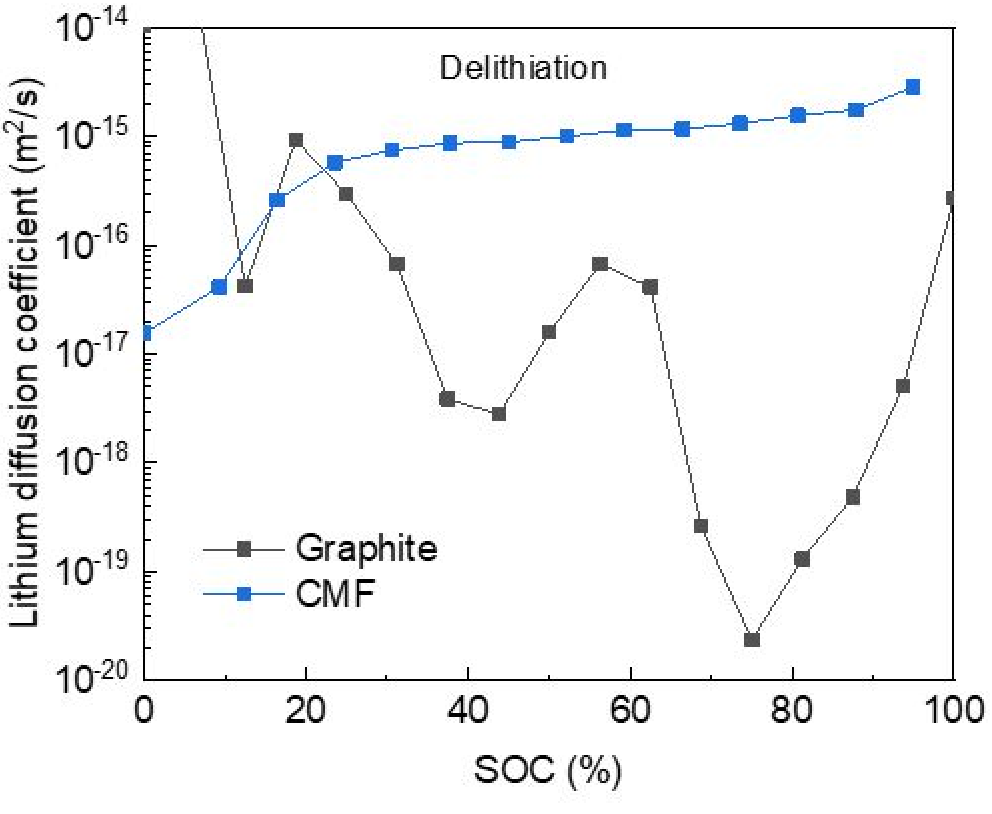
Delithiation
Graphite
CMF
Lithium diffusion coefficient (m2/s)
SOC (%)
10-14
10-15
10-16
10-17
10-18
10-19
10-20
0
20
40
60
80
100

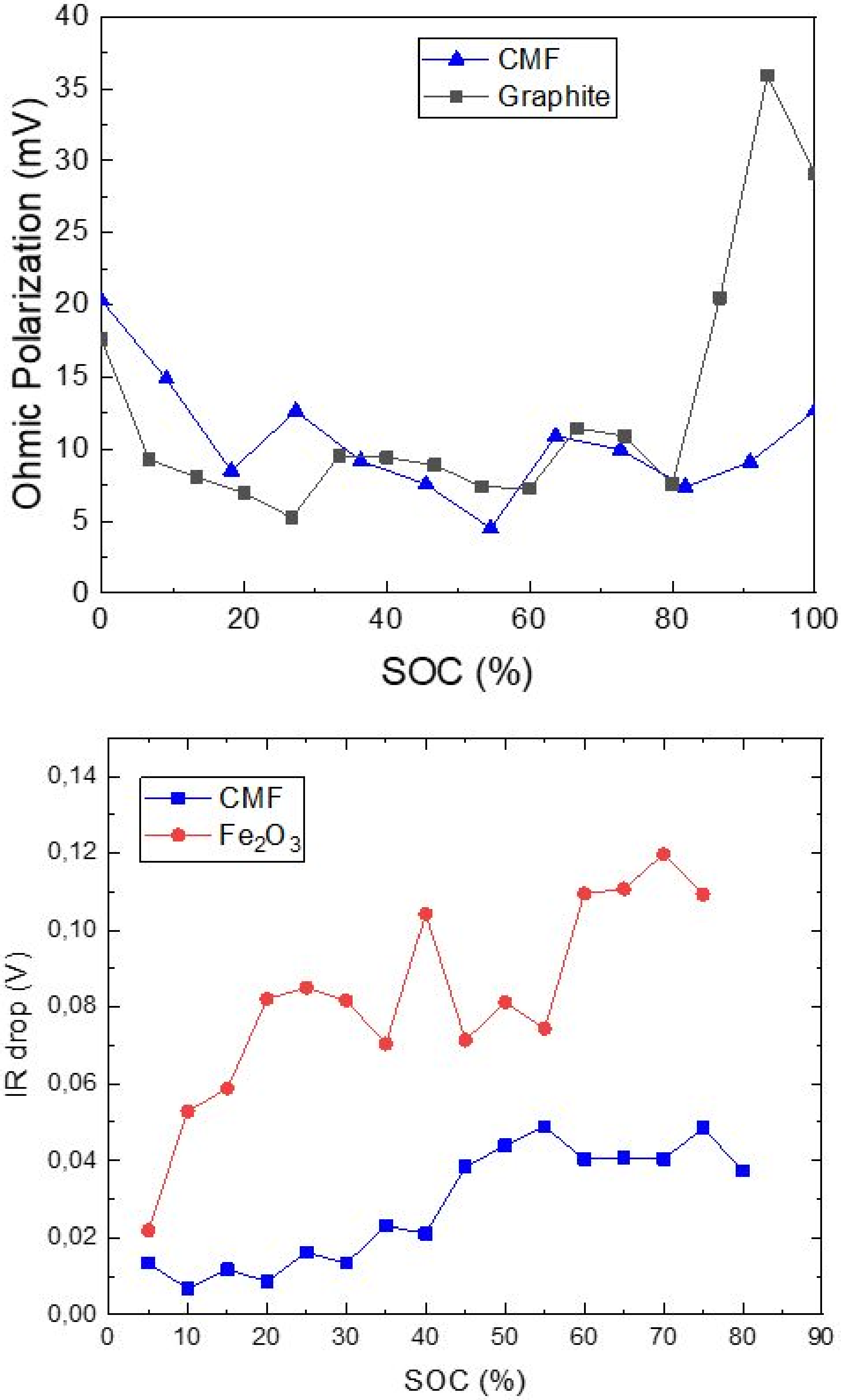


**Figure S1**. GITT experimental results.

# CMF Images

A digital photo and 3D XCT re-constructed images of CMF are shown in Figure S2.

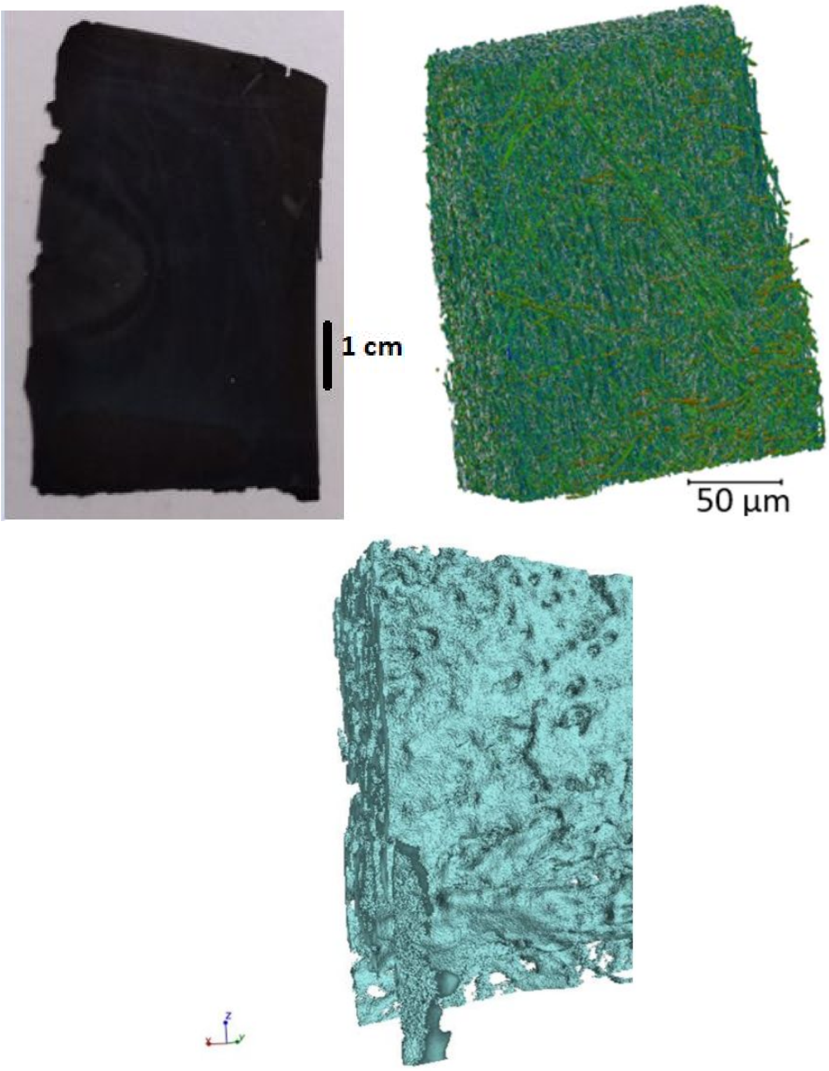


**Figure S2**. Carbon metal fabric elecrode images.

We specifically performed this bending process on a tissue paper so that any visible residue left on the tissue paper after bending could be observed (Figure S3). The CMF material that has not been bent is shown in the photo labeled (1). Bending and flexing can be seen in the photos labeled (2) and (3). In the photo labeled (4), it can be particularly noted whether any residue remains on the tissue paper after bending.

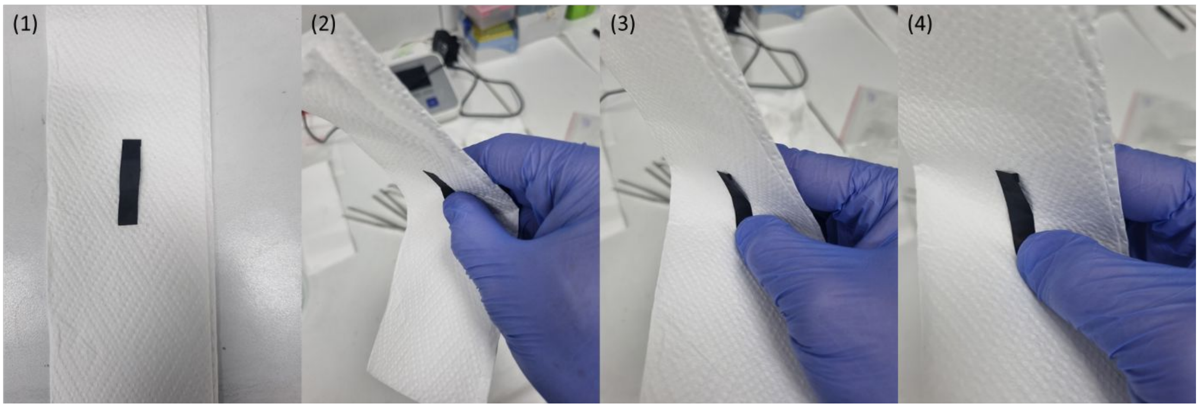


**Figure S3**. Digital images showing the flexibility of the CMF and minimal to nil material loss upon bending.

# Lithium resistivities and diffusion coefficient from half-cell testing

EIS was performed on the CMF and graphite half cells in the discharged (delithiated) state, with the spectra shown in Figure S4. The EIS spectra were fitted using Zfit software, enabling the extraction of the values for ohmic (R1) and charge transfer (R2) resistances (Table S1).

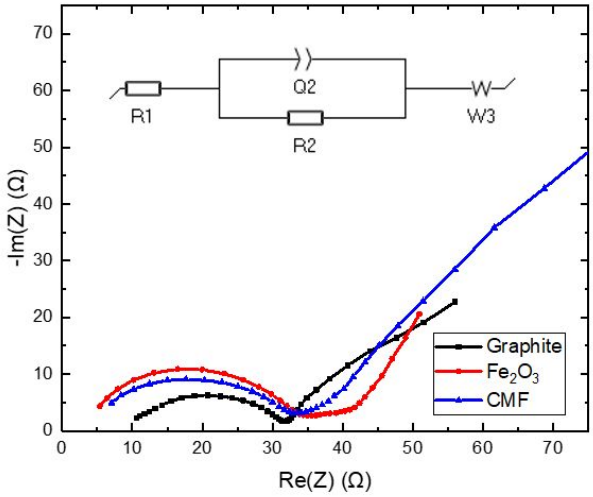


**Figure S4**. Resistivities of CMFs and the controls by means of EIS. An equivalent circuit with a control phase element (represented as Q2) is also shown.

**Table S1**: Parameters extracted from EIS spectra from half-cell testing.

| | CMF | Graphite | $Fe_2O_3$ |
|---|---|---|---|
| R1 (Ω) | 3.5 | 10.5 | 4.0 |
| R2 (Ω) | 28.4 | 25 | 30.5 |
| R3 (Ω) | 0 | 36 | 10.5 |
| $R_{total}$ (Ω) | 31.9 | 71.5 | 45.0 |
| $D_{Li+}$ ($m^2/s$) | $2.54 \times 10^{-18}$ | $7.06 \times 10^{-19}$ | $5.05 \times 10^{-19}$ |

Li diffusion characteristics can also be examined using the EIS spectra. In the low frequency region, a straight line is typically visible, relating to Warburg diffusion. Again, using Zfit software, values for the Warburg Impedance coefficient ($\sigma_w$) were extracted, and then used the calculate the diffusion co-efficient using the equation:

$$D_{Li^+} = \frac{R^2T^2}{2A^2n^4F^4C^2\sigma_w^2}$$

where $D_{Li+}$ is the $Li^+$ diffusion co-efficient, R is the gas constant (8.31 J $mol^{-1}$ $K^{-1}$), T is the temperature (293 K), A is the electrode area (1.77 $cm^2$), n is the number of electrons involved in the redox process (1), F is Faraday's constant (96,485 C $mol^{-1}$), C is the $Li^+$ concentration in the electrode material (0.0315 mol $cm^{-3}$), $\sigma_w$ is the Warburg Impedance (Ω $s^{-0.5}$) co-efficient calculated from impedance spectra. The calculated values of $D_{Li+}$ for CMF, $Fe_2O_3$ and graphite half cells are shown in Table S1. These values are two orders of magnitude lower than as measured by GITT (at 0 % SOC as shown in Figure S1). Large variations in $D_{Li+}$ measured using the two variations is common, nevertheless the values indicate enhanced diffusion within the CMF material compared with graphite and $Fe_2O_3$ slurry cast electrodes.

# Full-cell LIB coin cell testing

Figure S5 only shows the relative capacities as against the very initial value determined. Therefore, it is not a real indicator of the true performance of the LIB system but only a relative one. The LNMO||CMF combination appears to be advantageous as the high cathode voltage enables a good cell voltage (average discharge voltage is 3.70 V) and consequently a higher energy density. The LFP||CMF performed better at higher cycling rates (up to 50 C) and demonstrated greater capacity retention (not absolute but relative to the initial value) while undergoing repeated cycling at 1 C. The greater achievable C-rate in the full-cell configuration (compared with half-cell, above) was attributed to the elimination of the impedance associated with the Li foil used in half cell testing. It is expected that optimisation of the electrolyte for the CMF will further enhance full cell performance. The greater achievable C-rate in the full-cell configuration (compared with half-cell, above) was attributed to the elimination of the impedance associated with the Li foil used in half cell testing.[1] Additionally, the impedance of LFP-based full cell was lower than that of LNMO. It seems that the impedance values of half-cells in Figure S4 were lower than those in Figure S5, which could be explained by the fact that the replacement of Li ion chips by slurry cast cathode materials increases cell resistances.

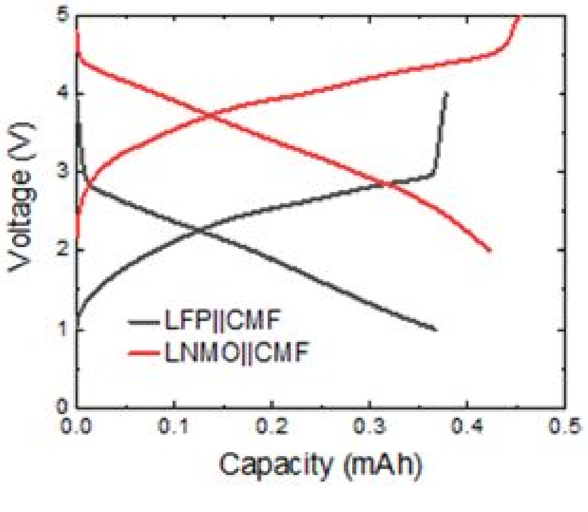

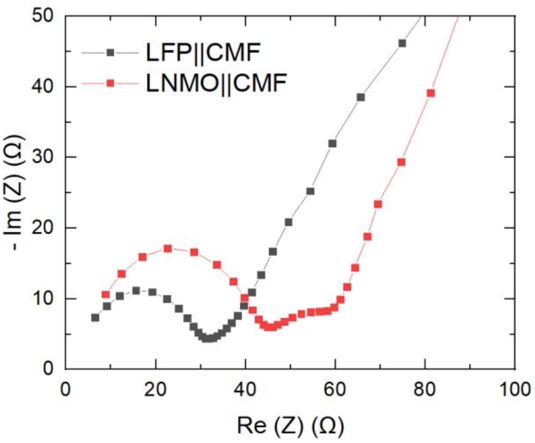


**Figure S5**. CMF anode along with two standard cathodes in a full lithium ion coin cell.

## Reference


(1) Geaney, H.; Bree, G.; Stokes, K.; McCarthy, K.; Kennedy, T.; Ryan, K. M. Highlighting the Importance of Full-Cell Testing for High Performance Anode Materials Comprising Li Alloying Nanowires. *Journal of The Electrochemical Society* **2019**, *166* (13), A2784. DOI: 10.1149/2.0291913jes.
